# Barriers and facilitators to cardiopulmonary resuscitation within pre-hospital emergency medical services: a qualitative study

**DOI:** 10.1186/s12873-021-00514-3

**Published:** 2021-10-13

**Authors:** Nahid Dehghan-Nayeri, Hassan Nouri-Sari, Fatemeh Bahramnezhad, Fatemeh Hajibabaee, Mojtaba Senmar

**Affiliations:** 1 Department of Critical Care and Nursing Management, School of Nursing and Midwifery, Tehran University of Medical Sciences, Nosrat St, Tehran, 1419733171 Iran; 2 Disaster and Emergency Medical Management Center, Ministry of Health and Medical Education, Tehran, Iran

**Keywords:** Cardiopulmonary resuscitation, Emergency care, Prehospital, Cardiac arrest, Qualitative research

## Abstract

**Background:**

Out-of-hospital cardiopulmonary arrest is a common and fatal problem. Rescuing patients with this problem by pre-hospital emergency medical services is associated with various barriers and facilitators. Identifying these barriers as well as the facilitators in a qualitative and an information-rich way will help to improve the quality of performing the maneuver and to increase the patients’ survival. Therefore, the current study was qualitatively conducted with the aim of identifying the factors affecting the cardiopulmonary resuscitation within the pre-hospital emergency medical services.

**Methods:**

This qualitative study was conducted using a content analysis approach in Iran in 2021. The participants were 16 Iranian emergency medical technicians who were selected through a purposive sampling method. For data collection, in-depth and semi-structured interviews were conducted. For data analysis, the Elo and Kyngäs method was applied.

**Results:**

The mean participants’ age was 33.06 ± 7.85 years, and their mean work experience was 10.62 ± 6.63 years. The collected information was categorized into one main category called “complex context of the cardiopulmonary resuscitation” and 5 general categories with 17 subcategories. These categories and subcategories include patient condition (patient’s underlying diseases, age, high weight, number of children, and place of living), dominant atmosphere in companions at home (companions’ feeling of agitation, companions doing harm, and companions helping), policy (educational policy, human resource policy, up-to-date equipment and technology, and do-not-resuscitate policy), performance of the out-of-organizational system (disorganization in the patient handover process, and cooperation of the support organizations), and conditions related to the treatment team (conscience, cultural dominance, and shift burden).

**Conclusions:**

The results showed that the conditions related to the patient and his/her companions, as well as the organizational factors such as the policies and the out-of-organizational factors act as the barriers and the facilitators to the cardiopulmonary resuscitation within pre-hospital emergency medical services. Therefore, the barriers can be modified and the facilitators can be enhanced by taking various measures such as educating, human resource policy-making, upgrading the equipment, and considering appropriate management policies.

**Supplementary Information:**

The online version contains supplementary material available at 10.1186/s12873-021-00514-3.

## Introduction

Out-of-hospital cardiopulmonary arrest is a major public health problem with high mortality rate [1, 2]. The survival of patients with this problem depends on a series of coordinated measures. This survival chain consists of community, hospital-based services, and rapid deployment of an emergency physician and ambulances [3]. Although cardiopulmonary resuscitation is the first- and the main- line treatment for the cardiopulmonary arrest [4], the survival rate of the out-of-hospital cardiopulmonary arrest varies between 5 and 38%, and this difference is related to the way that the pre-hospital emergency medical services are managed [5]. Improving the quality as well as the effectiveness of care for the patients with the out-of-hospital cardiopulmonary arrest is addressed as one of the tasks of the emergency medical services throughout the world; however, these systems differ significantly in terms of structure and organization [5].

Pre-hospital emergencies play a vital role in providing timely care to patients [6]. Although three decades have only passed since the establishment of the emergency medical system in Iran, there is plenty of evidence that in the short term, valuable services to the health system [7, 8]. Currently, more than 1000 students with Associate and 500 students with Bachelor’s degree in medical emergency graduate from Iranian universities of medical sciences. Nearly 14,000 individuals provide pre-hospital emergency services as technicians in Iran [9]. One of the goals of this system is to provide appropriate treatment in the right place and at the right time using available resources. In this system, the care starts from the time the patient calls and continues until the patient is delivered to the hospital [10]. The pre-hospital emergency personnel, as the front-line forces of providing pre-hospital services [11], arrive on the scene earlier than the other healthcare groups [12].

The pre-hospital emergency personnel should be able to make the fastest decision about transferring the patient to the medical centers and selecting the equipment needed for the transfer based on the judgment, the skill and the priority [13]. These personnel witness the critical condition of the patients, and experience time constraints as well as various stresses that will affect the quality of their work. It is vivid that this effect is, in fact, a threat to the health of the community and the lives of the residents of the area covered by the relevant emergency bases [14]. Therefore, the faster, more accurately and more correctly the patients are cared for at this stage, the lower the mortality and the higher the people’s trust in these systems [10]. The cardiopulmonary resuscitation is no exception to this rule, and the personnel face various barriers and facilitators during this maneuver.

In order for a team to perform a successful cardiopulmonary resuscitation, many factors such as the team members and the equipment must be properly aligned [15]. The fact is that the hidden aspects of the barriers and the facilitators to the cardiopulmonary resuscitation within the pre-hospital emergency medical services are unknown or little unknown, and have not been well studied. Since the method used in qualitative research is proposed as a suitable method for in-depth and comprehensive study of social phenomena, and it helps to study the phenomenon in a real and comprehensive way when the systematic information is not available, qualitative research is the best approach to identify these barriers and facilitators [16]. Various studies on the cardiopulmonary resuscitation have been conducted within pre-hospital emergency medical services; yet, very few study has qualitatively explored the barriers and the facilitators to this maneuver. Considering the different communities’ cultural, social and economic differences and the pre-hospital emergency services’ differences throughout different countries, the current study was conducted with the aim of identifying the factors affecting the cardiopulmonary resuscitation within the pre-hospital emergency medical services using a content analysis approach.

## Methods

### The aim, design and setting of the study

In reporting this qualitative study, we have adhered to the COREQ (Consolidated Criteria for Reporting Qualitative research) guidelines. In qualitative research, the content analysis approach is widely used to interpret the context-based data [17]. Therefore, in the current study, the Elo and Kyngäs method was applied. This method is recommended for phenomena about which limited information is available [18].

### Study participants

Samples in qualitative studies tend to be small to increase the depth of analysis [19]. The concept of saturation is the most important factor in determining the sample size in qualitative studies. Data saturation refers to the time that the data collection process no longer provides any new or relevant data [20]. In the current study, data saturation was obtained by conducting 13 interviews. However, in order to increase the depth and richness of information, 3 supplementary interviews were conducted with three extra participants. In addition, three participants were interviewed twice. On the whole, 16 participants were selected from pre-hospital emergency personnel using a purposive sampling method. Maximum variation sampling was used to achieve information richness and extensive information. Participants’ work shift varies between 175 and 300 h per month. During each work shift, the participants experience between 6 and 15 missions. The participants met the inclusion criteria if they had different professional backgrounds, at least 1 year of work experience, and experience of caring for 4 cases with the cardiopulmonary resuscitation during the last 2 months. The time and the place of the interviews were selected based on the participants’ opinions and in a quiet place without any noises.

### Data collection and analysis procedures

A total of 19 face-to-face and semi-structured interviews were conducted with all the participants. Each interview lasted between 45 and 90 min. The main questions included: What has been your experience with the cardiopulmonary resuscitation? What barriers and facilitators have you experienced during this maneuver? Please explain more about the barriers and the facilitators to this maneuver. During the interviews, in order to explore more about the participants’ beliefs and experiences, some exploratory questions were asked. All the interviews were recorded using a digital device. As soon as each interview finished, the data was typed in Microsoft Word. Data analysis was performed in the following steps: open coding, indexing, grouping, categorizing and abstracting. Accordingly, through the analysis process, the analysis units, which can be analyzed and coded, were selected from the interviews, and were categorized into smaller units including the meaning units, the codes (which include the title of coding for the meaning units), the categories (which include a group of content with meaning and conceptual commonality), and the main category.

### Trustworthiness

The quality criteria used in quantitative research, such as internal validity, generalizability, reliability, and objectivity, are not appropriate for judging the quality of qualitative research. Qualitative researchers speak of trustworthiness [21]. Several definitions and criteria of trustworthiness or rigor exist [22]. In this regard, Guba and Lincoln criteria including credibility, dependability, confirmability, transferability and authenticity were used [23, 24]. In this regard, the researcher devoted sufficient and appropriate time to each interview, and considered prolonged engagement with the participants to gain their trust. The interviews and the findings were reviews with the research team, and a summary of each participant’s findings was reviewed with the participant. The analysis of the data was provided in a detailed and in-depth way for the readers. The data and the categories were continuously reviewed and compared in terms of similarities and differences. For transferability, in-depth descriptions of the data were provided.

### Ethical considerations

The current study was approved by the Ethics Committee (code of ethics: IR.TUMS.FNM.REC.1398.125). All the participants were informed about the objectives and the methods of the study, their rights and the researchers’ duties. The confidentiality and the participants’ rights to continue or withdraw from the study were mentioned. The permission to record the interviews was obtained from the participants. After collecting the necessary data, the participants signed the informed consent form. The interviews were conducted until data saturation was reached.

## Results

Sixteen participants with the age range of 24–50 years participated in the current study. The minimum work experience was 2 years and the maximum was 29 years. The data analysis revealed 1 main category, 5 general categories and 17 subcategories (Table 1).

**Table 1:** the main, general and sub-categories of complex context of the cardiopulmonary resuscitation

Main category	General categories	Sub-categories
complex context of the cardiopulmonary resuscitation	patient condition	patient’s underlying disease
		patient’s age
		high weight of the patient
		having a child
		place of living
	dominant atmosphere in companions at home	companions’ feeling of agitation
		companions doing harm
		companions helping
	policy	education policy
		human resource policy
		up-to-date equipment and technology
		do-not-resuscitate policy
	performance of the out-of-organizational system	disorganization in the patient handover process
		cooperation of the support organizations
	conditions related to the treatment team	conscience
		cultural dominance
		shift burden

### The main category: complex context of the cardiopulmonary resuscitation

The cardiopulmonary resuscitation in pre-hospital emergency medical services is associated with various barriers and facilitators, so that these factors have led to the development of a complex context for this maneuver. For our participants, the barriers and the facilitators had several meanings which affected their performance and reaction.

### General category 1: patient condition

Patient condition is an immediate condition which it is related to the patient. In this general category, the subcategories play both the facilitating and the inhibiting roles in the cardiopulmonary resuscitation.

#### Patient’s underlying diseases

According to our findings, more than two-thirds of the participants believed that the presence of an underlying disease acts as a facilitator to the cardiopulmonary resuscitation. In their perspective, the presence of an underlying disease leads to a stress-free performance of the resuscitation. Regarding the presence of an underlying disease, the personnel, in addition to maintaining their true performance, do not get involved with the patient’s companions.

Participant No. 14 stated: *“I had a patient who had cancer and was taking medicine every day, and her death was near. During the time of the cardiopulmonary resuscitation, I was performing the task easily, and without any pressure and stress. I didn’t also get involved with the companions, because they had already known what was going on.”*

Based on the participants’ experiences, the patient’s underlying disease had a psychological impact on the patient’s family so that they can accept the patient’s death easier. In addition, the patient’s underlying disease along with calmer companions reduces stress in personnel.

#### Patient’s age

On the one hand, the participants expressed that the patient’s increasing age is a facilitator to the cardiopulmonary resuscitation. On the other hand, the patient’s decreasing age causes them to make more efforts. Fifteen out of the sixteen participants stated that this maneuver can change from the beginning to the end as the patient’s age changes.

Participant No. 12 stated: *“The first patient I had was under 50 years old, and we tried and performed the cardiopulmonary resuscitation for 45 minutes without any interruption. If the patient is younger, I feel that I should try harder. The second patient I had was over 80 years old. We tried, but you know, the patient had lived enough. We did our job calmly and without any stress.”*

It is clear that the personnel’s effort is never reduced depending on the patient’s age. However, patient’s increasing age is a facilitator by reducing their stress and the strain of cardiopulmonary resuscitation.

#### High weight of the patient

Participants expressed that they were unable to move those patients who were obese. Although the high weight of the patient leads to uninterrupted interventions, from the perspective of almost 66% of them, it plays as a barrier since it causes them from not being able to transfer the patient to a medical center with more equipment and staff.

Participant No. 13 stated: *“I think the patient weighed twice as much as me and three times as much as my colleague. My colleague came to me and said: “Let’s transfer him.” I said: “Who can do it? You or me?” We then performed all the interventions there. It is true that we did everything for him, but he was heavy and we couldn’t transfer him to a better place with more equipment and staff.”*

Based on the participants’ experiences, the high weight of the patient causes the personnel from not being able to transfer the patient to specialized centers with more staff. In addition, the high weight of the patient may even cause a waste of time in moving and transferring the patient.

#### Having a child

The participants expressed that they try harder if the patient with the cardiopulmonary arrest has a child. This effort even increases when the patient’s child is young. This can lead to more effort and motivation during the maneuver through having a positive effect on the personnel’s energy and emotions. It is worth saying that having no child does not reduce their effort. From the perspective of 10 participants, having a child is a facilitator to this maneuver.

Participant No. 9 stated: *“I saw that the patient had two young children. All I could see was the children. They gave me energy, and I told myself to try harder maybe the pulse returned.”*

Having a child seems to increase the feeling of empathy in the personnel and this can be considered as a facilitator.

#### Place of living

Some participants described that the patient’s place of living can play as a facilitating role in the cardiopulmonary resuscitation, because it has an absolute role in the amount of pressure and stress on the personnel. Fifteen participants expressed that in the lower areas of the city, they experience less pressure and stress and in the upper areas (areas often prosperous and have a new urban texture) of the city, they experience more psychological burden due to some conditions.

Participant No. 5 stated: *“The patient lived in the lower areas of the city, so I did my job easier. There was no stress that wanted to be relieved, so I did my job perfectly.”*

It seems that the cardiopulmonary arrest in the lower areas of the city, similar to the presence of an underlying disease, acts as a facilitator by reducing staff stress.

### General category 2: dominant atmosphere in companions at home

All of the participants explained that the dominant atmosphere in companions at home has a significant impact on their performance. Based on the participants’ experiences, this has both the visible and the hidden effects on the cardiopulmonary resuscitation. The atmosphere created by the companions during the cardiopulmonary resuscitation plays an important role in managing the situation, and the personnel’ set of actions and reactions. For instance, when the companions become agitated and want to attack the personnel, this atmosphere becomes unsuitable and it causes an interference in the cardiopulmonary resuscitation. If the companions help, this atmosphere will play as a facilitating role. This general category consists of three subcategories: the companions’ feeling of agitation, companions doing harm, and companions helping.

#### Companions’ feeling of agitation

All 16 participants stated that the presence of agitated companions disrupts the cardiopulmonary resuscitation. Based on their experiences, this, in addition to disrupting the maneuver, results in more complexity of the context of the cardiopulmonary resuscitation and acts as a barrier.

Participant No. 10 stated: *“At first, when the patient died, his daughters started crying and shouting a lot. We didn’t know whether to resuscitate the patient or to calm them down. I couldn’t do anything at that time. I couldn’t perform the cardiopulmonary resuscitation with all those shouting and offending. No one would go out so that I could do my job. At the end, when the atmosphere calmed down, we did our job.”*

Not only does the presence of agitated companions increase the tension on the personnel, but also calming them down doubles the work burden and prevents the correct cardiopulmonary resuscitation.

#### Companions doing harm

All of the participants believed that one of the barriers to the cardiopulmonary resuscitation is the companions doing harm since the personnel had to get involved with it. Companions doing harm in the form of disrespect, inappropriate reactions and beatings can be addressed as one of the significant barriers to this complex context.

Participant No. 6 stated: *“He followed us with a knife or dirk. I had completely forgotten everything. I didn’t know what to do now, and at that moment, I didn’t even know what the cardiopulmonary resuscitation was. We referred to a patient to do his works, but I was waiting for someone to come and say something to us. Whenever his companions came, they offended us. Come on … let me do my job. And at the end, they followed us with a knife.”*

It is clear that companions doing harm prevents proper cardiopulmonary resuscitation. The significant point is that the personnel in this situation not only lose their proper function, but also the cardiopulmonary resuscitation is interrupted and will not be practically useful.

#### Companions helping

The participants said that they had experienced a variety of contributions through the cardiopulmonary resuscitation. Based on the experience of 100% of the participants, this, in addition to being a facilitator to this maneuver, can also lead to improved services in this operation.

Participant No. 1 stated: *“I rode with two of the patient’s companions and I told one of them to do the bag-valve-mask ventilation and the other one to hold my knees so that I could give the massage. That was how I could continue the massage, and no disturbances occurred in the blood circulation.”*

It is clear that any help from the companions at any stage of cardiopulmonary resuscitation is a facilitator to this maneuver. It should be noted that this can lead to continued cardiopulmonary resuscitation and increase the likelihood of patient survival.

### General category 3: policy

This category indicates that the design and the implementation of a set of policies regarding education, human resource, up-to-date equipment and technology, and do-not-resuscitate can play a prominent role in the cardiopulmonary resuscitation. All of the 16 participants expressed that the appropriate consideration of these policies plays as a facilitating role, and non-consideration or a disturbance in the consideration of these appropriate policies has an inhibiting role in the cardiopulmonary resuscitation.

#### Educational policy

The current policy is that the personnel must attend in-service and continuing education classes in 1 year and complete at least 70 h of related education per year. Based on our findings, 100% of the participants believed that the implementation of appropriate educational policy will improve the quality of the cardiopulmonary resuscitation. In the participants’ perspective, the implementation of this policy by the pre-hospital emergency medical services has led to adequate education of the personnel, and the people and the personnel’s reactions have changed from the time when these policies did not exist. This subcategory expresses the appropriate policy in a category that affects the whole resuscitation maneuver and acts as a facilitator to the maneuver. The appropriate effect of the education on the preparation to resuscitate as well as the improvement of the education were the relevant policies.

Participant No. 7 stated: *“Anyway, before going to the class, there were a series of questions that I didn’t know the answers for them and they were all in my mind. When I went to the class and saw the scenes, I found the answers to these questions and I didn’t have those questions anymore. I knew the answers to my questions, and my mind was ready for any situation. In that cardiopulmonary resuscitation scene, I was no longer involved in what I should do now. I had learned everything in the class and I had implemented them. In the beginning, it wasn't like this, and I was getting really confused in the scenes. Our continuous education wasn’t enough. Within the last few years, some changes have taken place and I think they pay a great attention to the education.”*

It seems that the implementation of appropriate educational policy can be a facilitator to cardiopulmonary resuscitation through facilitating the personnel’s ability and updating their information. In addition, some experiences indicated that this subcategory also increases personnel’s confidence.

#### Human resource policy

Human resource is one of the most significant factors affecting the cardiopulmonary resuscitation. The current policy is that only two forces are deployed for all the cardiopulmonary resuscitation maneuvers, and these two should perform all the relevant maneuvers and measures. Considering a policy of increasing the number of forces by sending motor lances and ambulances at the same time directly affects the maneuver and the personnel’s reactions and the measures, and facilitates this maneuver. On the contrary, all of the 16 participants expressed that the lack of human resource or sending only two forces for performing the cardiopulmonary resuscitation is considered as a barrier to the cardiopulmonary resuscitation.

Participant No. 3 stated: *“During that operation, we were really few in number. We had a shortage of human resource. How can two people resuscitate a patient for 45 minutes? It isn’t at all reasonable to do a complete and correct maneuver by two forces. Eventually, you either have to get tired or try less. But, if we were 3 or 4, we could do better. Like the time that they sent a motor lance with us at the same time to help that boy. There was no extra pressure and the maneuver was performed more accurately.”*

The interview revealed that if the policy of increasing the personnel in cardiopulmonary resuscitation is adopted, fatigue will be reduced and cardiopulmonary resuscitation will be associated with better results.

#### Up-to-date equipment and technology

Based on the experiences of all of the 16 participants, the use of compatible and appropriate technologies by the system has a significant role in reducing their workload. The participants believed that apart from the appropriate and up-to-date technology, the use of an alternative and faster transportation system is one of the most significant policies that can have positive effects on the cardiopulmonary resuscitation. Considering approaches such as improving the equipment in recent years and introducing the Asayar automation system to accelerate the cardiopulmonary resuscitation durations have been among the appropriate policies of the system that act as facilitators to this maneuver.

Participant No. 3 stated: *“In terms of equipment, after the arrival of the physician, because of the political relations and policies which have been made, the situation has improved a lot. Almost 70% of emergency bases across the country have electroshock devices. During the last few years, the Asayar (Electronic registration system) automation system has been launched that has saved a lot of time and resources for pre-hospital emergency medical services.”*

Based on the findings, it can be said that the provision of up-to-date equipment and technology reduces the personnel’ workload in this maneuver. In addition, considering that there are two people for this maneuver, it makes it easier for them.

#### Do-not-resuscitate policy

In Iran, the do-not-resuscitate order has not been legally approved, and besides, because of the religious beliefs, several problems are associated with it. From the perspective of 66% of the participants, the lack of the do-not-resuscitate order is a barrier to this maneuver. They attribute the lack of this order to some patients’ ineffective and unreasonable efforts and performance.

Participant No. 12 stated: *“I think there should be a do-not-resuscitate order so that it can protect me, the technician. Now the blood money (*If a patient dies due to intentional or unintentional staff misconduct, personnel are required to be financially and non-financially responsible by the law) *for a person is 2000 to 3000 million Rials (*Approximately $8,000 to $12,000)*, his/her companion can easily report and put me in a trouble. So, it is good. It decreases our tiredness, saves equipment and facilities, and will provide us with a legal protection. For this, there was a patient that we went by to perform the cardiopulmonary resuscitation. All the companions told us not to resuscitate their patient, but we didn’t have a legal thing called the do-not-resuscitate order. So, we had to ineffectively resuscitate the patient for 45 minutes.”*

Based on the experience of the participants, the do-not-resuscitate order can facilitate this maneuver by reducing workload, fatigue and providing legal protection. In addition, this order can reduce the cost and resource loss in some patients.

### General category 4: performance of the out-of-organizational system

Within the cardiopulmonary resuscitation operations, after sending an ambulance to the scene, if necessary, the police and fire brigade will also be sent especially in cases such as suspected cardiopulmonary arrest and hanging. The last stage for this operation is the delivery of the patient with cardiac massage and artificial respiration to the hospital. Thus, the cardiopulmonary resuscitation can be influenced by a concept called the performance of the out-of-organizational system. Since this maneuver requires the cooperation and coordination of a set of factors, the most significant of which is the out-of-organizational system. These systems can act as both a facilitator and a barrier. Based on our findings, all of the participants believed that the coordination and cooperation of the out-of-organizational system has a facilitating role in cardiopulmonary resuscitation and the lack of this coordination and cooperation acts as a barrier to it. A disorganization in the patient handover process, and the cooperation of the support organizations are subcategories of this category.

#### Disorganization in the patient handover process

In some missions, the patient, due to his/her condition and vital signs, needs to be taken to the hospital along with advanced life support. The participants expressed that they witness and experience some disorganizations in this process, which they had a negative impact on the quality of the cardiopulmonary resuscitation and staff interaction. Therefore, from the perspective of all of the 16 participants, this disorganization acts as a barrier to the cardiopulmonary resuscitation.

Participant No. 8 stated: *“The hospital servants didn’t help us transfer the patient from the emergency bed to the hospital bed. This isn’t our duty. We transferred the patient ourselves, and we ourselves moved him. During the transfer, we couldn’t resuscitate the patient and the maneuver was interrupted. We shouted for help so that they can help us move the patient, but nothing happened. We no longer had energy. We were tired, and we got tired even more. We no longer had energy for the next cardiopulmonary resuscitation. We even had no energy to go.”*

Based on the findings, disorganization in the patient handover process causes a waste of time, interruption of continuous cardiopulmonary resuscitation and increased fatigue in personnel. These cases lead to this subcategory being considered as a barrier to this maneuver.

#### Cooperation of the support organizations

The cardiopulmonary resuscitation scene is a special scene full of complexity. In this scene, due to the created conditions, the presence of support organizations is sometimes needed in order to improve the maneuver. It can be said that this maneuver is not only affected by the healthcare organizations, but also by other cooperating and relief organizations. All of the participants believed that the cooperation of support organizations such as the fire department and the police station plays a significant facilitating role in the cardiopulmonary resuscitation.

Participant No. 5 stated: *“I remember the time that we went by to help a patient with the cardiopulmonary arrest. When the patient died, if there were no police there, we were done (*This means that we could be harmed)*. In my opinion, the police should be present in all the cardiopulmonary resuscitation scenes. They help a lot so that we can manage the scene. They control the companions and don’t allow them to approach the patient, so we can at least examine the patient.”*

It can be said that the cooperation of the support organizations not only creates psychological peace in the personnel, but also help them manage the cardiopulmonary resuscitation scene.

### General category 5: conditions related to the treatment team

Within the cardiopulmonary resuscitation, the team sent to the scene has a number of features and conditions that can affect the entire operation. Based on data analysis, all of the 16 participants stated that conditions such as conscience, cultural dominance and shift burden can play as a facilitating and an inhibiting role in the cardiopulmonary resuscitation.

#### Conscience

The participants’ experiences revealed that the maneuver can be better performed if something called ‘conscience’ exists. The conscience had a facilitating role for them. The participants stated that from the beginning to the end of the maneuver, the conscience acts as a calming force and as an accelerating factor in the operation, and it leads to more effort. In other words, it can be said that for all of the 16 participants, the presence of conscience plays an important role during this maneuver.

Participant No. 7 stated: *“I tried to resuscitate the patient, at least because of my conscience. I went on a mission at every level and environment, but I conscientiously tried to work in such a way that not only this patient but also any other patient wouldn’t be different for me. These discriminations and injustices had no impact on my performance, and I conscientiously worked for the patient. If I came out of the base late and moved slowly, my conscience would hurt. My conscience doesn’t accept that I should leave the base late or move slowly because of ... I think the conscience always comes first.”*

What the experience shows is that the conscience is an esoteric understanding by the personnel since they have achieved it without any training. This factor can act as a facilitator to this maneuver by increasing the personnel’s energy and communication with God.

#### Cultural dominance

Based on the experience of all of the 16 participants, culture is an important factor in cardiopulmonary resuscitation. Culture refers to the customs that are common among the personnel, and they act in accordance with it. Some of these customs, although not formally and legally dictated, have been passed on informally between different generations of the personnel and have a significant effect on their function. The participants stated that some priorities were common among them and they acted in accordance with them. These priorities can play as a barrier or a facilitator in this maneuver. Another dimension of this concept is related to the position that the patient has for the personnel. In other words, if the patient’s position is good and the concept of being human is understood, this culture plays as a facilitating role in the maneuver. The participants said that some of their colleagues behave outside the rules and regulations, which leads to a disruption in the process and plays as an inhibiting role.

Participant No. 9 stated: *“In the emergency medical services, we have a priority that follows as: me, my colleague, the ambulance, and the patient. It has always been like this. During one mission, I observed these priorities. Because if I hurt myself, I couldn’t do the maneuver. If the ambulance had a problem, so be it. All of these made me follow the priorities, and then, get to the patient and perform the cardiopulmonary resuscitation.”*

Participant No. 4 stated: *“During one mission, one of my colleague did the maneuver in such a way that as if he/she was his own parents, and this itself caused him not to give up and work hard. However, during that mission of the young lady, who had a cardiac arrest, my colleague didn’t seem to care about these things, and he didn’t try much to bring the patient back to life.”*

Cultural domination seems to have two dimensions. In case of mastering a correct culture, we will see an increase in accuracy and quality, and in case of mastering an incorrect culture, we will sometimes see a waste of time and a decrease in the quality of this maneuver.

#### Shift burden

The average hours worked by a force is a minimum of 175 h and a maximum of 350 h per month. The participants explained that the multiple shifts as well as the consecutive shifts act as the barriers to the cardiopulmonary resuscitation. Besides, all of them stated that an increase in the work burden leads to a decrease in the quality of the cardiopulmonary resuscitation and plays as a barrier.

Participant No. 16 stated: *“I work 300 hours a month. It is obvious that there are a lot of pressure on me, and this can affect my performance. When I get to a patient, I am really tired. I work in the hospital for 12 hours and now I have to be in the pre-hospital emergency medical services for 24 hours. I don’t have the enough energy to perform the cardiopulmonary resuscitation at all. The truth is, since I don’t have the enough energy, I don’t try hard.”*

Based on the findings, it is clear that shift burden acts as a significant barrier to this maneuver through reducing energy, increasing workload and fatigue.

In general, we must state that facilitation and obstruction each have different dimensions that are affected by several factors. This facilitation or obstruction is greatly influenced by the judgment of the personnel, and each participant looks at these issues from his/her own perspective.

## Discussion

In this study, the experiences of the pre-hospital emergency personnel in relation to the barriers and the facilitators to the cardiopulmonary resuscitation were analyzed. The results indicated that there are several barriers and facilitators including the patient condition, the dominant atmosphere in companions at home, the policy, the performance of the out-of-organizational system, and the conditions related to the treatment team which have resulted in the development of one category called ‘the complex context for the cardiopulmonary resuscitation’. It is worth saying that no study has examined the barriers as well as the facilitators to the cardiopulmonary resuscitation within the pre-hospital emergency medical services.

The results of the current study showed that the presence of an underlying disease, increasing age, having a child, and the patient’s place of living are among the facilitators to the cardiopulmonary resuscitation. In addition, the high weight of the patient was a barrier to this maneuver by causing the personnel from not being able to transfer the patient to the hospital. The results of some studies showed that the presence of an underlying disease is one of the main barriers to a successful cardiopulmonary resuscitation [25, 26]. These studies indicated that the performing a successful cardiopulmonary resuscitation is lower in patients with an underlying disease and this condition is considered as a barrier. However, in the current study, the existence of an underlying disease from the perspective of personnel with reducing stress and creating peace of a facilitator was raised. The results of one study showed that the presence of an underlying disease has no effect on the success rate of the cardiopulmonary resuscitation [27]. This quantitative study investigated the effect of the presence of an underlying disease on the success rate of the cardiopulmonary resuscitation, while in the current study, only the implementation of the cardiopulmonary resuscitation was considered. Some studies showed that there is no relationship between the success rate of the cardiopulmonary resuscitation and age [27, 28]. These studies have quantitatively examined the survival of patients undergoing the cardiopulmonary resuscitation. However, in the current study, the survival of patients undergoing the cardiopulmonary resuscitation was not considered and we were only looking for the barriers and the facilitators to the cardiopulmonary resuscitation. The results of one study also showed that the rate of bystander’s cardiopulmonary resuscitation increases as the patient’s age decreases [29]. It can be said that on the one hand, reducing the patient’s age is a facilitator for the personnel, and on the other hand, increasing the patient’s age can be a barrier for the personnel so as to try harder. This finding is largely in line with the results of the current study. One study showed that nurses in rural areas face several challenges [30]. Although this study was not about the cardiopulmonary resuscitation, it seems that living in rural areas, given the conditions, plays as an inhibiting factor for care, which in terms of inhibition, it is somewhat in line with the results of the current study. However, no study was found to investigate the effect of the patient’s place of living on the cardiopulmonary resuscitation. Another study showed that the body mass index is not independently associated with the patient’s survival rate [31]. This quantitative study investigated the output of the cardiopulmonary resuscitation, and its setting and research method are different from the current study. However, the patient’s weight does not seem to be a barrier or a facilitator, and therefore, it is not in line with the current study. In another study, morbid obesity was an independent predictor for increased in-hospital cardiopulmonary resuscitation mortality [32]. In this quantitative study, mortality in obese patients was worse than that for non-obese patients. It can be said that the obesity is a barrier to the successful cardiopulmoary resuscitation. Although the above study did not study the facilitating or inhibiting role of obesity, but to some extent, it can be said that the obesity can be a barrier to the cardiopulmonary resuscitation. In one study, the rate of survival until discharge of the patients in different groups of body mass index, except for low weight people, was expressed similarly [33]. It can be said that in the above study, low weight facilitates the survival until discharge of patients. This finding is somewhat in line with the findings of the current study. No study was found to investigate the effect of the patient’s number of children on the performance of the cardiopulmonary resuscitation.

The results of the current study showed that the companions’ feeling of agitation and companions doing harm were among the barriers to the cardiopulmonary resuscitation, while the companions help was among the facilitators of the performance of this maneuver. The results of one study showed that loud talking, arguing, and aggression are some examples of disrespectful behaviors that nurses would experience [34]. The results of another study indicated the discussion with the cardiopulmonary resuscitation team as one of the subcategories of the destructive presence of the companions during the cardiopulmonary resuscitation [35], which it is somewhat in line with the findings of the current study. Companions doing harm can be presented in the form of physical and verbal violence. In this regard, some studies have reported the annual rate of experiencing various types of violence by the emergency medical technicians to be over 65% [24, 36], and the most common types of violence are verbal and physical violence [37–39]. These results indicate the existence of this kind of violence which it is somewhat in line with the findings of the current study. In terms of the companion helping, most studies examined the effects of the presence of the companions during the cardiopulmonary resuscitation and expressed the positive effects of this presence for the family. For instance, in one review study, one of the effects of this presence was to reduce the stress in family members [40], while the current study showed that the presence of the companions, at the end of the attendance spectrum, can help the personnel do their jobs.

In the current study, educational policy as well as the up-to-date equipment and technology were addressed as the facilitators. The results of one study showed that the personnel do not receive adequate education on the cardiopulmonary resuscitation in hemodialysis wards. This study demonstrated that the education acts as a facilitator to the cardiopulmonary resuscitation [41]. The results of other studies also showed the education as a facilitator [42, 43]. The results of these studies are in line with the findings of the current study. One study showed that the lack of equipment is one of the challenges of the pre-hospital emergency medical services [14], which it expresses the role of this matter as a facilitator. In other words, up-to-date equipment and technology can be considered as one of the facilitators to the cardiopulmonary resuscitation, which it is somewhat in line with the current study. The current study showed that providing enough human resource is one of the facilitators to the cardiopulmonary resuscitation. The results of one study showed that the lack of human resource as a challenge increases the workload and the stress within the cardiopulmonary resuscitation [44]. The results of one study showed that the shortage of human resource is one of the problems in the pre-hospital emergency medical services [14]. In other words, it indicated that considering an appropriate human resource policy plays as a facilitator. The current study showed that the lack of do-not-resuscitate order is one of the barriers to cardiopulmonary resuscitation. The results of one study stated that the lack of the do-not-resuscitate order is an ethical challenge in the cardiopulmonary resuscitation [44]. The results of one study showed that nurses have a positive perspective on the do-not-resuscitate order when the measures taken do not save the patients’ lives [45]. This finding is somewhat in line with the findings of the current study. In another study, doubts about the do-not-resuscitate order were not expressed as a barrier to the cardiopulmonary resuscitation [41], which it is not in line with the findings of the current study.

The results of another study showed that the lack of coordination and collaboration between the organizations is one of the major barriers in providing the services to patients [46]. The results of one study showed that more than 90% of the participants have difficulty in receiving the patients from the pre-hospital emergency medical services and delivering the patients to the hospital wards [47]. This finding is in line with the findings of the current study, and it suggests the disorganization in the patient handover process. The results of other studies are demonstrating this problem as well [48, 49]. Another study found that hospital staff do not cooperate well [50]. This finding is in line with the findings of the current study. Contrary to the findings of the current study, one study showed that the personnel face a lack of proper cooperation and coordination from different organizations such as the police in traffic accidents [50]. It is true that this finding is not in line with the experiences of the participants of the current study, yet the facilitating role of these organizations is discussed. Hence, it can be said that this finding is somewhat in line with the findings of the current study.

In line with the findings of the current study, the results of one study indicated the roles of caring, blaming and ethics for the conscience [51]. The results of another study stated that the conscience is the cause of doing the right thing without the presence of an external supervision [52]. The results of another study showed that the work ethic is one of the factors in improving the quality of nursing care [53]. All of these findings emphasized the facilitating role of the conscience, which it is in line with the current study. The results of one study identified the cultural barriers as one of the barriers to the implementation of evidence-based practice [54]. Another study showed that habits mediate the planning and the professional behavior of the healthcare providers [55], which it is somewhat in line with the findings of the current study. The results of one study indicated that one of the challenges of providing emergency services during the early stages is the high workload [56], which it is in line with the results of the current study.

## Conclusions

The findings of this study showed that the complex context of the cardiopulmonary resuscitation within the pre-hospital emergency medical services is associated with several barriers and facilitators. Therefore, in order to provide patient-centered services, the necessary opportunities should be provided to acquire clinical knowledge and skills to improve the situation. The policymakers are expected to implement programs such as increasing personnel, providing more equipment, using up-to-date technology, educating people, increasing coordination with other organizations, and providing financial and psychological support to personnel in order to provide the appropriate conditions for better and more appropriate implementation of the cardiopulmonary resuscitation. It is suggested that in future studies, the barriers and the facilitators to the cardiopulmonary resuscitation be examined particularly in different areas. The results are not generalizable, but they can be transmitted. These results can be applied by the researchers for those participants which their context is similar to the current study.

## Supplementary Information

**Additional file 1: Table S1.** The participants’ demographic characteristics.

## Data Availability

Data are available by contacting the corresponding author.

## Abbreviations

EMS: Emergency Medical Service
CPR: Cardiopulmonary Resuscitation
TUMS: Tehran University of Medical Sciences
OHCA: Out of Hospital Cardiac Arrest
