# Supplementary material for: Barriers and facilitators to cardiopulmonary resuscitation within pre-hospital emergency medical services: a qualitative study
Source: BMC Emerg Med. 2021 Oct 13;21:120. doi: 10.1186/s12873-021-00514-3 (PMC8515705; doi:10.1186/s12873-021-00514-3)
Supplement: Supplementary file 1 — Additional file 1: Table S1. The participants’ demographic characteristics. [file 12873_2021_514_MOESM1_ESM.docx]

**Table 1.** the participants’ demographic characteristics

| No. | sex | age | work experience | units |
| --- | --- | --- | --- | --- |
| 1 | male | 27 | 2 | road, urban base |
| 2 | male | 32 | 8 | road, urban base |
| 3 | male | 30 | 10 | road, urban base |
| 4 | male | 34 | 9 | road, urban base |
| 5 | male | 30 | 9 | road, urban base |
| 6 | male | 28 | 10 | road, urban base |
| 7 | male | 38 | 14 | road, urban base |
| 8 | female | 24 | 2 | triage, the navigation unit |
| 9 | male | 35 | 13 | road, urban base |
| 10 | male | 50 | 15 | road, urban base |
| 11 | male | 50 | 29 | road, urban base |
| 12 | male | 32 | 12 | road, urban base |
| 13 | male | 24 | 4 | road, urban base |
| 14 | male | 34 | 13 | road, urban base |
| 15 | female | 36 | 16 | triage, the navigation unit |
| 16 | female | 25 | 4 | triage, the navigation unit |
